# Economic Crisis and Amenable Mortality in Spain

**DOI:** 10.3390/ijerph15102298

**Published:** 2018-10-19

**Authors:** Andreu Nolasco, Pamela Pereyra-Zamora, Elvira Sanchis-Matea, Nayara Tamayo-Fonseca, Pablo Caballero, Inmaculada Melchor, Joaquín Moncho

**Affiliations:** 1 Research Unit for the Analysis of Mortality and Health Statistics, Department of Community Nursing, Preventive Medicine, Public Health and History of Science, University of Alicante, Campus de San Vicente del Raspeig s/n, Ap. 99-03080 Alicante, Spain; nolasco@ua.es (A.N.); elvirasanchis@hotmail.es (E.S.-M.); nayara.tamayo@ua.es (N.T.-F.); pablo.caballero@ua.es (P.C.); melchor_inm@gva.es (I.M.); joaquin.moncho@ua.es (J.M.); 2 Mortality Register of the Valencian Community, section of Epidemiological Studies and Health Statistics, General Sub-directorate of Epidemiology and Health Monitoring, General Directorate of Public Health, Health Ministry of the Valencian Government, 03010 Alicante, Spain

**Keywords:** mortality, amenable mortality, economic recession, socioeconomic factors, Spain

## Abstract

*Background:* Both overall mortality and avoidable mortality have decreased in recent years in most European countries. It has become clear that less privileged socioeconomic groups have an increased risk of death. In 2008, most countries went into a severe economic recession, whose effects on the health of the population are still ongoing. While on the one hand, some evidence associates the economic crisis with positive health outcomes (pro-cyclical effect), on the other hand, some other evidence suggests that the economic crisis may pose serious public health problems (counter-cyclical effect), which has given rise to controversy. *Objectives:* To describe the evolution of overall mortality and amenable mortality in Spain between 2002–2007 (before the economic crisis) and 2008–2013 (during the economic crisis), nationally and by province, as well as to analyse trends in the risks of death and their association with indicators of the impact of the crisis. *Methods:* Ecological study of overall mortality and amenable mortality describing the evolution of the risks of death between 2002–2007 and 2008–2013. Age Standardised Rates were calculated, as well as their percentage change between periods. The association between percentage changes and provincial indicators of the impact of the crisis was analysed. Amenable mortality was studied both overall and categorised into five groups. *Results:* Amenable mortality represented 8.25% of overall mortality in 2002–2007, and 6.93% in 2008–2013. Age Standardised Rates for overall mortality and global amenable mortality generally declined, with the sharpest decline in amenable mortality. Decreases in overall mortality and amenable mortality were directly related to vulnerability indicators. The most significant decreases were registered in ischaemic heart disease, cerebrovascular disease, and other amenable causes. The relationship with vulnerability indices varied from direct (cancer) to inverse (hypertensive disease). *Conclusions:* Amenable mortality shows a more significant decrease than overall mortality between both study periods, albeit unevenly between provinces causes of death. Higher vulnerability indicators entail greater declines, although this trend varied for different causes. Mortality trends and their relationship with socioeconomic indicators in a situation of crisis must be conducted cautiously, taking into consideration a possible pro-cyclical effect.

## 1. Background

Avoidable mortality (AM) is usually considered as a key indicator for revealing health inequalities, i.e., unfair differences in health quality, welfare, and healthcare provision based on social, geographical, ethnic, educational, gender or other differences. This includes differences in the presence of the disease, health results or access to health services [1]. According to the type of health prevention intervention, AM may be of two types:

(a) Preventable mortality (PM): primary prevention provided by health services, lifestyle, intervention programmes.
(b) Amenable mortality (AMM): secondary prevention, i.e., access to healthcare, advising, diagnosis or treatment provided by health services [2,3].

Socioeconomic indicators are related to AM, and particularly to AMM, due to a higher mortality rate among the less privileged, as has been pointed out in several studies conducted in different countries [2,4,5,6,7,8]. These inequalities are a risk factor for the health of the population.

In Spain, AMM shows an even sharper declining trend than overall mortality (OM) [2,9,10]. The same declining trend applies in other European countries, though other lists of causes and study periods were considered [4,11].

Specifically, AMM may be influenced by a number of factors, such as healthcare benefits, geographical location, and other socioeconomic factors [1]. Identifying geographical areas (provinces, regions…) with higher risks of AMM would allow to carry out specific actions in health aimed at reducing mortality and, thus, inequalities [5].

Generally speaking, a steady improvement in living standards and a better access to healthcare have contributed to a decline in AMM [2,5,11,12,13]. Nevertheless, a number of studies have proved that this trend is related to socioeconomic indicators of inequality, thereby relating less privileged groups to higher levels of mortality [2,5,7,8,14,15].

The recession that began in 2008 has affected, to a greater or lesser extent, most western countries and many others, and has substantially modified the production and distribution of goods and services [16,17]. Impacts of the 2008 severe economic recession on the health of the population are still underway. Research is also developing in differing ways. Although some evidence associates the economic crisis with positive health outcomes (pro-cyclical effect), some other evidence suggests that the economic crisis affects negatively on public health (counter-cyclical effect). Research on the effects of the recent economic crisis on health is therefore still in progress [18]. Thus, further research is needed to determine the evolution of health during the period of economic crisis, during which the living conditions of many sectors of the Spanish population have undoubtedly become deteriorated and certain changes in health services that hinder access and affect the quality of health services have been introduced.

In Spain, to date, no research on AMM trends in the periods under consideration and their relationship with socioeconomic indicators has been conducted. As a result, there is a need to assess the effects of the current economic crisis on AMM and socioeconomic inequalities, since the policies applied by the Spanish government and the European Union aimed at overcoming the crisis may be crucial to reduce or increase possible impacts on the health of the population.

The objective of this study is to describe the evolution of the risks of OM and AMM between 2002–2007 and 2008–2013, both nationally and by province, as well as to analyse provincial trends in the risks of death and their association with socioeconomic indicators of the impact of the crisis.

## 2. Materials and Methods

### 2.1. Type of Study and Data Sources

This is an ecological study of OM and AMM. Annual mortality figures occurred to residents in Spain during the study period (2002–2013) by sex, age, province of residence, and cause of death were obtained from the Spanish National Statistics Institute. Population figures by age (five-year age groups) and sex were also obtained from the Spanish National Statistics Institute. As a measure of the socioeconomic impact of the crisis, four indices have been considered for each province, based on the percentage evolution of fifteen indicators between 2006 and 2013: Economic Vulnerability Index (evolution of the economic activity and employment, on the basis of five indicators: GDP per capita, number of companies, employed population, unemployed population, and foreign trade), Social Vulnerability Index (socio-demographic changes, on the basis of five indicators: population and immigration, residential mobility, recipients of unemployment benefits, young people emancipation, and social mobilization), Real-Estate Vulnerability Index (evaluates the evolution of the real estate market, based on another five indicators: number and value of mortgages, empty completed dwelling, real-estate dwelling transactions, average price of empty dwelling, and mortgage foreclosures), and composite Index of Total Vulnerability (ITV), consisting of an average of the previous three indices, based on a standardised construction process (range of values: −2, lowest vulnerability; 2, highest vulnerability) (see Supplementary Table S1). Further details on the process of construction and values of the aforementioned indicators may be found in Méndez et al. [19]. To analyse OM, all deaths were considered jointly regardless of the cause of death. The causes of AMM and age ranges included in this study are the ones suggested by Nolte and McKee (see Table 1), including 50% of deaths by ischaemic heart disease [12,20].

Our data are secondary data extracted from the Register of Mortality. Therefore, these data are not subjected to ethical considerations as ethical considerations were taken into account by this authority.

The mortality data used in this study can be downloaded from the webpage of the Spanish National Statistics Institute and the total vulnerability index by province is available from the book *Atlas de la crisis. Impactos socioeconómicos y territorios vulnerables en España* by Ricardo Méndez et al. [19].

### 2.2. Analysis Methods

For the analysis of AMM, all deaths were considered jointly, and grouped into five groups: cancer (causes 7 to 14), ischaemic heart disease (cause 20), cerebrovascular disease (cause 21), hypertensive disease (cause 19), and other amenable causes (see Table 1).

Age Standardised Mortality Rates for overall mortality (ASR) and amenable mortality (AASR) have been calculated for each study period by sex, both nationally and for the 52 Spanish provinces. The national population of Spain during the study periods (the sum of the populations during the years studied) was used as the standard population. Annual rates were also calculated at a national level.

For each unit of analysis, the percentage decrease of ASR and AASR for the second vs. the first period was calculated as (1 − (ASR2008-13/ASR2002-07)) × 100 and (1 − (AASR2008-13/AASR2002-07)) × 100. We calculated 95% confidence intervals (95% CI). This decrease quantifies the relative decrease in standardised rates from the first to the second period.

Basic descriptive indicators of ASR and AASR, and the relative decrease in OM and AMM have been calculated on the basis of provincial indicators: mean, standard deviation, minimum value, maximum value, and coefficient of variation. The coefficient of variation of rates and relative decreases was considered as the provincial measure of inequality for the corresponding indicator.

In order to analyse the relationship between the relative decrease in mortality and provincial socioeconomic indicators of the impact of the crisis, Spearman’s Rank Correlation Coefficient was used. The significance level applied was 0.05.

## 3. Results

The total number of deaths during the study periods was 2,255,761 in 2002–2007 and 2,323,380 in 2008–2013. Among men, the total number of deaths was 1,175,548 in the first period and 1,194,579 in the second period. Among women, it was 1,080,213 in the first period and 1,128,801 in the second period. A slight increase in OM can be noted among both men and women (see Supplementary Table S2).

AMM represented 8.25% of OM in the period 2002–2007 and 6.93% in the period 2008–2013 in both sexes. Among men, 106,282 (9.04%) of the total deaths were due to amenable causes in the first period, and 91,368 (7.65%) in the second period. Among women, 79,863 (7.39%) of the total deaths were due to amenable causes in the first period, and 69,539 (6.16%) in the second period. A slight decline in AMM was observed between both periods among both men and women.

Mortality rates by province, sex, and type of cause (OM and AMM) for the study periods are available in supplementary Table S2. The trends in ASR and AASR in Spain for the study period, showed a steady decrease among both men and women (see Supplementary Figure S1).

Table 2 shows the statistical descriptions of standardised mortality rates and the relative decreases of these, calculated on the basis of the values available for each province. At a national level, Spain’s decrease in overall mortality standardised rate (ASR) is 14.43% among men and 12.48% among women. The decrease in amenable mortality standardised rates (AASR) is 19.26% among men and 17.54% among women.

The coefficient of variation of standardised rates, a relative variability indicator, is in all cases higher for total AMM than for OM. It declines slightly from the first to the second period for OM, whereas it increases among men but decreases among women for AMM. It is to be highlighted that AMM shows greater declines and higher coefficients of variation than OM, which indicates greater variability and thus inequality.

The results vary on the basis of the different groups of amenable causes of death studied. While ischaemic heart disease, cerebrovascular disease, and other amenable causes show the greatest decrease in mortality (higher than 20% in all cases), similarly distributed among men and women, the decrease for cancer was barely noticeable among men (0.79%) and small among women (8.14%). Concerning hypertensive disease, different results were obtained for men and women. It increased among men (−2.26% percentage decrease), but decreased significantly among women (16.75%).

The results disaggregated by sex can be found in Supplementary Tables S3 (men) and S4 (women). These tables contain the total OM and AMM standardised rates, as well as the relative decreases and 95% confidence intervals by province for the periods 2002–2007 and 2008–2013.

Table 3 shows Spearman’s Rank Correlation Coefficients between the indicators of the impact of the crisis and the relative decreases in ASR and AASR. It can be observed that the composite index of total vulnerability shows moderate correlations, which are significant regarding both OM and AMM (except for amenable mortality among men). Higher (worse) vulnerability entails higher decrease in mortality. When observing these correlations disaggregated by components of the index, it is noted that the result is similar for economic and real-estate vulnerability, but not for social vulnerability, where correlations are low and non-significant in all cases (*p* < 0.05).

In the analysis of these correlations by group of causes, no significant associations were found between any of the causes analyzed with the index of total vulnerability in men. Only a statistically significant inverse correlation was evidenced for the case of hypertensive diseases and the social component of the index.

On the other hand, in women, significant associations have been observed in mortality due to cerebrovascular diseases with a higher value of index of total vulnerability and with the indices of Real-Estate Vulnerability and Economic Vulnerability. In addition, there was a significant direct association in the case of other avoidable deaths with the Social Vulnerability Index.

## 4. Discussion

This paper examines changes in the risks of death by any cause or by amenable avoidable causes between 2002–2007 (before the crisis) and 2008–2013 (during the crisis) in Spain. It has been proved that national and provincial ASR and AASR in Spain decreased between the study periods, with a greater decline in AASR. Inequalities between Spanish provinces were higher for amenable mortality. Disparities in the declines of the rates were greater for amenable mortality. Concerning total OM and AMM, the analysis showed in general an inverse and significant relationship with economic and real-estate vulnerability, but not with social vulnerability. Nonetheless, the analysis of the correlations by group of amenable causes showed a differential trend.

The declining trend in AMM found in this study is similar to the one described in other papers [2,9,10,21,22], which suggest that this decline may be due to prevention measures taken for risk factors and the development of treatments and technologies [9], as well as the entry into force of Law 42/2010 on health measures against the use of tobacco and regulating the sale, supply, consumption, and advertising of tobacco [23,24,25].

This trend varies depending on the group of causes of AMM. Among men, cancer and hypertensive disease barely varied between both periods, while ischaemic heart disease, cerebrovascular disease, and other amenable causes show a great decrease. Among women, all groups decrease but the pattern is similar to the one for men: greater decline in ischaemic heart disease, cerebrovascular disease, and other causes; and lower decrease in cancer and hypertensive disease. A recent study has described mortality trends in Spain before and during the Great Recession. Using mortality data from 2004 to 2011, this study found that the OM rate declined more during the first four years of the crisis period (2008–2011) than in the four years preceding the crisis (2004–2007), especially in low socioeconomic groups, with similar results for different specific causes, such as cardiovascular diseases, except for cancer in women [26].

Additionally, in 2011 the acute myocardial infarction rate was reduced among men under [27]. Several studies have proved the relationship between the implementation of laws on the use of tobacco and the decrease in the risk of cardiovascular and respiratory diseases in the short term, and cancer in the long term) [27].

The results obtained show geographical inequalities between provinces both in mortality rates and in their decline between periods (higher in AMM than in OM). Other studies have proved the existence of geographical inequalities in mortality between Spanish provinces [28,29,30,31,32]. Existing literature suggests that these inequalities may be related to the political management of the crisis carried out in each of the provinces, which could have influenced its impact on the health of the population [18,20,33].

These general results match the ones obtained in some other studies, which prove a greater decrease in mortality during periods of crisis [16,34]. The positive correlation between the impact of the crisis and the decrease in both OM and AMM seems to verify the paradox that periods of economic slowdown may bring beneficial effects on health, which gives rise to the pro-cyclical theory. The mechanisms that explain this inverse relationship may vary depending on the cause of death and changes in risk factors, such as the decrease in environmental pollution due to industrial production [35], decline of stress caused by working environments due to higher unemployment [36,37], increase of social benefits, improved lifestyle [35], decrease in tobacco sales [24], etc. In support of this theory, a report released by the Spanish Health Ministry reflects an increased positive assessment of self-perceived health in the Spanish population between 2006 and 2011 [38]. This indicator is a short-term predictor of mortality and the use of health services [39,40], since a better self-perceived health would entail a decrease in the risk of death. Other effects contributing to this pro-cyclical theory might derive from the implementation of law 42/2010 on the control of tobacco consumption, which led to a significant decrease in cigarette consumption and sale between 2011 and 2012.

Concerning mortality by cancer, the results in this paper do not coincide with the ones recently described by Maruthappu et al. [41] in a study carried out on a large population sample, which describes a counter-cyclical effect of mortality by cancer during periods of crisis, strongly associated to unemployment. Nevertheless, this same paper also suggests that having access to universal public health (as in the case of Spain) may protect against this effect. This would be compatible with the sense of the observed correlations regarding this cause in our study, even though no statistical significance could ever be proved.

The results obtained for mortality from ischemic heart disease and cerebrovascular disease were also compatible with a procyclical relationship with the vulnerability index, although they were only statistically significant in women and for cerebrovascular diseases. Nonetheless, other studies have proved an increase in the risks of death in periods of crisis. Thus, factors such as malnutrition, substandard housing, the rise of mental health disorders, drug addiction, longer wait lists [42], and evictions and energetic poverty [43] increase the risk of death, giving rise to the so-called counter-cyclical theory. Unemployment is also considered as part of this theory by some authors [44], since the group of unemployed may suffer from worsened health indicators (for instance cardiovascular risk) as a consequence of the stress generated by an uncertain working situation and low security in periods of economic crisis.

Regarding mortality due to hypertensive disease, the results suggest a countercyclical effect, especially in men. Factors associated with the crisis may complicate the control of hypertension: On the one hand, anxiety and stress situations lead to an increase in conditions of this type; on the other hand, they promote both non-compliance with the treatments proposed by the doctors and neglecting healthy lifestyles.

It should be borne in mind that the economic crisis may have different effects on the short and long term. The results obtained in the study may be influenced by a potential delay in the harmful effects of the crisis. Although the results indicate that decreases in mortality are generally associated to worse vulnerability indices, the study period might be considered short. Thus, it is deemed necessary to conduct a new study in the future, since these results may be substantially modified in the medium and long term, as it is reasonable to think that the effects on chronic health problems, disabilities, and some of the main causes of death will be observed much later than other health results [1,5,12,45]. It should be taken into account, as highlighted by other authors [46], that the consequences of the crisis in the short, medium and long term in terms of social exclusion, unemployment, and poverty or economic cuts, such as medicine co-payment, which could be preventing some retirees from buying necessary drugs, or the decrease in doctors’ working hours due to restrictions in professional replacements, could have a negative impact.

This study has some limitations. Firstly, it is an ecological study; therefore, no cause-effect association can be drawn from it. Regarding the causes of death analysed, the list used could have been different. The choice was based on comparability criteria with other studies. The construction of indicators of the impact of the crisis was conceived on the basis of non-arbitrary and solid criteria, although it is true that in some cases the grouping of the indicators is doubtful; for instance, mortgage foreclosures have a real-estate basis but are an obvious social problem; employment is an economic activity indicator but also has a social impact. The criteria used have been reasoned and offer a three-dimensional view of the territorial impact of the crisis that is open to improvement and clarification.

## 5. Conclusions

Amenable mortality shows a greater decrease than overall mortality between the study periods. Declines were uneven between provinces and groups of amenable causes. Ischaemic heart disease, cerebrovascular disease, and other amenable causes show a higher decrease. Greater decreases were associated to worse vulnerability indicators, although when specifying by cause this trend was variable. It is concluded that the analysis of mortality trends and their relationship with socioeconomic indicators in periods of crisis must be conducted cautiously, taking into account possible pro-cyclical quantitative effects. It is necessary to analyse further each specific cause or group of causes. The effects detected could be modified in the medium or long term. The sustainment of a universal health system may decisively contribute to the decrease in mortality in periods of economic crisis.

## Figures and Tables

**Table 1:** ICD-10 classification code and age range for the amenable causes studied.

	Cause	ICD-10	Age
1	Intestinal infections	A00-09	0–14
2	Tuberculosis	A15-A19, B90	0–74
3	Other infections (diphtheria, tetanus, poliomyelitis)	A36, A35, A80	0–74
4	Whooping cough	A37	0–14
5	Septicaemia	A40-A41	0–74
6	Measles	B05	1–14
7	Malignant neoplasm of colon and rectum	C18-C21	0–74
8	Malignant neoplasm of skin	C44	0–74
9	Malignant neoplasm of breast	C50	0–74
10	Malignant neoplasm of cervix uteri	C53	0–74
11	Malignant neoplasm of cervix uteri and body uterus	C54-C55	0–44
12	Malignant neoplasm of testis	C62	0–74
13	Hodgkin’s disease	C81	0–74
14	Leukaemia	C91-C95	0–44
15	Diseases of the thyroid	E00-E07	0–74
16	Diabetes	E10-E14	0–49
17	Epilepsy	G40-G41	0–74
18	Chronic rheumatic heart disease	I05-I09	0–74
19	Hypertensive disease	I10-I13, I15	0–74
20	Ischaemic heart disease: 50% of deaths	I20-I25	0–74
21	Cerebrovascular disease	I60-I69	0–74
22	All respiratory diseases	J00-J09, J20-J99	1–14
23	Influenza	J10-J11	0–74
24	Pneumonia	J12-J18	0–74
25	Peptic ulcer	K25-K27	0–74
26	Appendicitis	K35-K38	0–74
27	Abdominal hernia	K40-K46	0–74
28	Cholelithiasis and cholecystitis	K80-K81	0–74
29	Nephritis and nephrosis	N00-N07N17-N19N25-N27	0–74
30	Benign prostatic hyperplasia	N40	0–74
31	Maternal death	O00-O99	All
32	Congenital cardiovascular anomalies	Q20-Q28	0–74
33	Perinatal deaths	P00-P96, A33, A34	All
34	Misadventures to patients	Y60-Y69, Y83-Y84	All

ICD: International Statistical Classification of Diseases.

**Table:** (**a**)

	Overall Mortality			Amenable Mortality (Total)		
	ASR ^$^ 2002–2007	ASR 2008–2013	ΔASR ^#^	AASR ^$^	AASR	ΔAASR ^#^
**Men**						
Spain	958.23	819.92	14.43	81.84	66.08	19.26
Provinces						
Average	944.79	819.32	13.24	81.18	66.81	17.61
Standard deviation	90.86	77.97	2.26	12.13	10.22	4.54
Minimum value	770.14	681.12	8.36	61.83	51.97	3.91
Maximum value	1140.38	997.28	18.96	112.2	95.15	26.20
Coefficient of variation (%)	9.61	9.52	17.07	14.94	15.3	25.78
**Women**						
Spain	860.37	753.03	12.48	59.58	49.13	17.54
Provinces						
Average	861.24	758.64	11.83	60.03	49.95	16.35
Standard deviation	99.31	83.83	2.35	10.9	8.27	5.63
Minimum value	719.62	632.16	6.48	42.07	36.53	−1.51
Maximum value	1104.90	1024.91	16.96	94.68	79.99	29.50
Coefficient of variation (%)	11.53	11.05	19.87	18.16	16.56	34.43

ΔAASR ^#^ (% decrease) by group of amenable causes.

**Table:** (**b**)

	Cancer	Ischaemic Heart Disease	Cerebrovascular Disease	Hypertensive Disease	Other Causes
	(Causes 7 to 14)	(Cause 20)	(Cause 21)	(Cause 19)	
**Men**					
Spain	0.79	25.84	29.07	−2.26	22.65
Provinces					
Average	−3.41	23.88	26.84	−8.24	22.15
Standard deviation	19.42	46.62	6.84	33.66	9.48
Minimum value	−123.50	6.83	12.08	−156.76	−1.12
Maximum value	27.39	9.79	38.11	66.67	45.68
Coefficient of variation (%)	569.50	28.60	25.48	408.49	42.79
**Women**					
Spain	8.14	30.69	28.90	16.75	22.03
Provinces					
Average	6.07	29.28	25.03	13.98	22.28
Standard deviation	8.20	12.78	13.95	28.84	10.62
Minimum value	−17.26	−23.41	−35.51	−84.91	−4.95
Maximum value	22.86	163.43	43.87	60.40	49.97
Coefficient of variation (%)	135.10	43.65	55.73	206.29	47.67

^$^ ASR = Overall Mortality Age Standardised Rate; AASR = Amenable Mortality Age Standardised Rate. Standard Population: The sum of the populations during the period 2002–2013. ^#^ ΔASR = Percentage decrease in ASR between 2002–2007 and 2008–2013; ΔAASR = Percentage decrease in AASR between 2002–2007 and 2008–2013.

**Table 3:** Spearman’s rank correlation coefficient ^$^ between the indicators of the impact of the crisis and the relative decreases in overall and amenable mortality.

Cause of Death		Real-Estate Vulnerability	Economic Vulnerability	Social Vulnerability	Total Vulnerability
**Men**					
Overall mortality	Rho	0.441 *	0.502 *	0.017	0.468 *
	Sig.	(0.001)	(0.001)	(0.904)	(0.001)
Total amenable mortality	Rho	0.166	0.323 *	0.053	0.242
	Sig.	(0.239)	(0.020)	(0.708)	(0.084)
Cancer (causes 7 to 14)	Rho	0.162	0.231	0.111	0.204
	Sig.	(0.252)	(0.100)	(0.433)	(0.147)
Ischaemic heart disease	Rho	0.135	0.096	−0.192	0.061
(cause 20)	Sig.	(0.340)	(0.500)	(0.173)	(0.666)
Cerebrovascular disease	Rho	0.045	0.134	0.066	0.115
(cause 21)	Sig.	(0.754)	(0.343)	(0.642)	(0.417)
Hypertensive disease	Rho	−0.122	0.011	−0.281 *	−0.149
(cause 19)	Sig.	(0.388)	(0.938)	(0.044)	(0.290)
Other causes	Rho	0.015	0.160	0.006	0.078
	Sig.	(0.916)	(0.256)	(0.964)	(0.581)
**Women**					
Overall mortality	Rho	0.360 *	0.340 *	0.102	0.382 *
	Sig.	(0.009)	(0.014)	(0.473)	(0.005)
Amenable mortality	Rho	0.445 *	0.295 *	−0.087	0.350 *
	Sig.	(0.001)	(0.034)	(0.538)	(0.011)
Cancer (causes 7 to 14)	Rho	0.232	0.194	0.210	0.257
	Sig.	(0.097)	(0.168)	(0.134)	(0.066)
Ischaemic heart disease	Rho	0.130	0.174	−0.037	0.138
(cause 20)	Sig.	(0.358)	(0.218)	(0.795)	(0.330)
Cerebrovascular disease	Rho	0.473 *	0.316 *	−0.046	0.403 *
(cause 21)	Sig.	(0.001)	(0.022)	(0.745)	(0.003)
Hypertensive disease	Rho	0.054	0.126	−0.230	0.006
(cause 19)	Sig.	(0.704)	(0.372)	(0.100)	(0.966)
Other amenable causes	Rho	0.004	−0.064	−0.378 *	−0.133
	Sig.	(0.979)	(0.654)	(0.006)	(0.348)

^$^ In brackets: statistical significance value for zero correlation hypothesis testing. * Statistical significance (*p* < 0.05). Rho = Spearman’s rank correlation coefficient. Sig. = Significant.

## Supplementary Materials

The following are available online at http://www.mdpi.com/1660-4601/15/10/2298/s1. Table S1: Indices of economic impact of the crisis in Spain by province, Table S2: Frequencies of death by sex and group of causes (overall and amenable mortality). Spain 2002–2007 and 2008–2013, Table S3: Standardised mortality rates, relative decreases, and 95% confidence intervals, by province. Periods 2002–07 and 2008–13. Men, Table S4: Standardised mortality rates, relative decreases, and 95% confidence intervals, by province. Periods 2002–07 and 2008–13. Women, Figure S1: Overall and amenable mortality annual standardised rates for the study period in Spain (2002–2013).

## Abbreviations

AM	Avoidable mortality
PM	Preventable mortality
AMM	Amenable mortality
OM	Overall mortality
ASR	Age Standardised Mortality Rates for overall mortality
AASR	Age Standardised Mortality Rates for amenable mortality
