# Supplementary material for: Economic Crisis and Amenable Mortality in Spain
Source: Int J Environ Res Public Health. 2018 Oct 19;15(10):2298. doi: 10.3390/ijerph15102298 (PMC6211017; doi:10.3390/ijerph15102298)
Supplement: Supplementary file 1 [file ijerph-15-02298-s001.pdf]

Supplementary Material

Economic Crisis and Amenable Mortality in Spain

Andreu Nolasco [1], Pamela Pereyra-Zamora [1,*], Elvira Sanchís-Matea [1], Nayara Tamayo-Fonseca [1], Pablo Caballero [1], Inmaculada Melchor [1,2] and Joaquín Moncho [1]

[1] Research Unit for the Analysis of Mortality and Health Statistics, Department of Community Nursing, Preventive Medicine, Public Health and History of Science, University of Alicante, Campus de San Vicente del Raspeig s/n, Ap, 99-03080 Alicante, Spain; nolasco@ua.es (A.N.); elvirasanchis@hotmail.es (E.A.-M.); nayara.tamayo@ua.es (N.T.-F.); pablo.caballero@ua.es (P.C.); melchor_inm@gva.es (I.M.); joaquin.moncho@ua.es (J.M.)

[2] Mortality Register of the Valencian Community, section of Epidemiological Studies and Health Statistics, General Sub-directorate of Epidemiology and Health Monitoring, General Directorate of Public Health, Health Ministry of the Valencian Government, 03010 Alicante, Spain

Supplementary Table S1. Indices of economic impact of the crisis in Spain by province.

Province	Real-Estate Vulnerability Index	Economic Vulnerability Index	Social Vulnerability Index	Index of Total Vulnerability (ITV)
Alava	-1.40	0.00	-0.40	-0.60
Albacete	0.20	0.20	1.00	0.47
Alicante	0.80	1.80	-0.20	0.80
Almeria	1.20	1.20	0.20	0.87
Asturias	-0.20	0.40	-1.20	-0.33
Avila	1.00	0.60	0.00	0.53
Badajoz	-1.20	-1.20	-0.80	-1.07
Balears (Illes)	0.20	0.80	0.40	0.47
Barcelona	1.00	0.80	0.40	0.73
Burgos	0.60	0.20	0.60	0.47
Caceres	-1.40	-0.60	0.40	-0.53
Cadiz	0.60	0.80	0.20	0.53
Cantabria	0.20	0.80	-0.60	0.13
Castellón	1.40	2.00	0.40	1.27
Ceuta	-1.20	-0.40	0.40	-0.40
Ciudad Real	0.60	0.20	0.60	0.47
Cordoba	-0.20	-0.40	0.60	0.00
Coruña (A)	-0.20	-1.00	-0.20	-0.47
Cuenca	-0.20	-0.40	0.40	-0.07
Girona	0.80	0.60	0.40	0.60
Granada	0.20	0.20	0.20	0.20
Guadalajara	1.60	0.40	-0.60	0.47
Guipuzcoa	-2.00	-0.60	-1.00	-1.20
Huelva	0.60	-0.20	-1.20	-0.27
Huesca	0.60	-0.60	-0.40	-0.13
Jaen	0.20	-0.40	0.00	-0.07

Table S1. *Cont.*

Leon	0.00	-0.20	1.00	0.27
Lleida	1.40	0.80	-0.20	0.67
Lugo	-1.20	-0.80	-1.40	-1.13
Madrid	0.20	0.20	-0.40	0.00
Malaga	0.80	0.80	0.60	0.73
Melilla	-0.60	-1.00	-0.40	-0.67
Murcia	1.40	1.20	0.60	1.07
Navarra	-1.20	-0.20	0.00	-0.47
Ourense	-1.20	-1.20	-0.80	-1.07
Palencia	-1.00	-0.60	-1.20	-0.93
Palmas (Las)	0.60	0.00	-0.40	0.07
Pontevedra	-0.20	0.20	-0.80	-0.27
Rioja (La)	0.80	0.60	0.20	0.53
Salamanca	0.00	-1.00	-0.20	-0.40
S.C. de Tenerife	0.60	0.20	-0.60	0.07
Segovia	-0.40	1.20	0.40	0.40
Sevilla	-0.20	0.20	-1.00	-0.33
Soria	-1.60	-0.40	0.40	-0.53
Tarragona	1.20	1.20	-0.20	0.73
Teruel	-1.20	-0.20	0.80	-0.20
Toledo	1.20	2.00	-0.60	0.87
Valencia	0.80	1.40	1.00	1.07
Valladolid	0.60	-0.60	0.00	0.00
Vizcaya	-1.80	-0.40	-0.40	-0.87
Zamora	-1.40	-0.40	-1.00	-0.93
Zaragoza	0.40	1.40	-0.20	0.53

Source: Méndez et al. "Atlas de la crisis. Impactos socioeconómicos y territorios vulnerables en España". Table 7.2.1 page 247 [19]. Reproduced with permission of the author.

Supplementary Table S2. Frequencies of death by sex and group of causes (overall and amenable mortality). Spain 2002-2007 and 2008-2013.

Province	Men				Women			
	Overall mortality		Amenable mortality		Overall mortality		Amenable mortality	
	2002-07	2008-13	2002-07	2008-13	2002-07	2008-13	2002-07	2008-13
**TOTAL SPAIN**	**1,175,548**	**1,194,579**	**106,282**	**91,368**	**1,080,213**	**1,128,801**	**79,863**	**69,539**
Alava	7,463	7,970	645	568	6,498	6,985	387	416
Albacete	10,296	10,752	795	642	9,401	10,013	611	507
Alicante	42,585	45,490	4,477	4,161	36,792	40,046	3,193	3,045
Almeria	14,492	15,115	1,457	1,347	12,143	13,115	1,160	999
Asturias	38,777	38,732	3,383	2,781	36,661	38,044	2,290	1,925
Avila	6,560	6,387	461	367	5,704	5,893	363	249
Badajoz	20,507	20,482	1,943	1,607	18,817	18,921	1,472	1,165

Table S2. *Cont.*

Balears (Illes)	22,829	23,989	1,927	1,839	20,534	22,372	1,547	1,497
Barcelona	133,758	133,433	11,321	9,780	127,369	131,912	8,648	7,622
Burgos	11,498	11,870	864	762	9,896	10,705	587	489
Caceres	13,310	13,436	1,166	919	11,852	12,140	813	658
Cadiz	27,959	29,230	3,288	2,885	24,346	26,054	2,554	2,297
Cantabria	16,922	17,183	1,364	1,223	15,403	16,339	950	855
Castellón	15,413	15,486	1,315	1,233	13,894	14,258	1,001	830
Ceuta	1,585	1,633	217	200	1,423	1,483	180	160
Ciudad Real	15,253	15,291	1,202	949	14,871	14,870	990	823
Cordoba	22,140	22,232	2,041	1,661	20,726	20,896	1,550	1,283
Coruña (A)	34,838	35,643	3,080	2,632	33,665	35,580	2,300	1,974
Cuenca	7,160	7,286	500	432	6,525	6,680	359	288
Girona	17,373	18,211	1,359	1,271	15,655	16,725	971	909
Granada	23,606	23,864	2,315	1,918	21,347	22,110	1,798	1,518
Guadalajara	5,387	5,709	379	344	4,855	5,101	282	245
Guipuzcoa	19,100	19,491	1,770	1,410	17,414	18,381	1,098	940
Huelva	13,306	13,324	1,367	1,230	12,294	12,555	1,129	963
Huesca	7,968	7,782	535	460	6,915	7,140	357	306
Jaen	19,410	19,702	1,788	1,432	17,471	17,735	1,338	1,060
Leon	17,867	17,986	1,315	1,058	16,066	16,736	934	783
Lleida	13,123	12,964	890	730	11,764	11,911	696	590
Lugo	15,326	15,254	1,180	937	14,023	14,191	784	570
Madrid	123,753	124,553	11,007	9,245	119,367	125,619	9,045	7,955
Malaga	35,560	37,003	3,896	3,617	31,132	33,009	3,018	2,684
Melilla	1,332	1,393	147	160	1,324	1,279	139	133
Murcia	30,852	32,236	3,045	2,698	27,610	29,462	2,362	2,112
Navarra	16,048	15,950	1,244	1,031	14,502	15,327	786	684
Ourense	14,123	13,945	988	885	13,543	13,802	710	582
Palencia	6,261	6,135	489	416	5,623	5,971	340	285
Palmas (Las)	20,660	21,687	2,401	2,112	16,132	17,530	1,807	1,583
Pontevedra	25,318	26,091	2,175	1,840	24,793	26,235	1,701	1,488
Rioja (la)	8,746	8,999	711	610	7,643	8,240	445	437
Salamanca	11,494	11,545	829	674	11,018	11,170	669	520
S.C. de Tenerife	21,009	22,047	2,283	1,990	17,553	19,162	1,663	1,637
Segovia	5,078	5,082	321	269	4,578	4,932	267	241
Sevilla	45,125	45,428	5,374	4,552	42,120	43,165	3,995	3,449
Soria	3,485	3,453	215	164	3,304	3,202	133	128
Tarragona	19,566	20,502	1,601	1,516	17,334	18,385	1,192	1,101
Teruel	5,417	5,413	398	271	4,767	4,746	262	202
Toledo	16,835	17,266	1,196	1,011	15,719	15,759	982	826
Valencia	65,619	65,659	6,342	5,528	61,000	62,944	4,780	4,188
Valladolid	14,054	14,228	1,211	1,077	12,855	13,586	922	800
Vizcaya	33,295	33,782	3,157	2,528	30,017	31,634	2,142	1,806
Zamora	7,876	8,056	563	463	7,448	7,541	402	306
Zaragoza	28,231	28,199	2,355	1,945	26,507	27,210	1,769	1,437

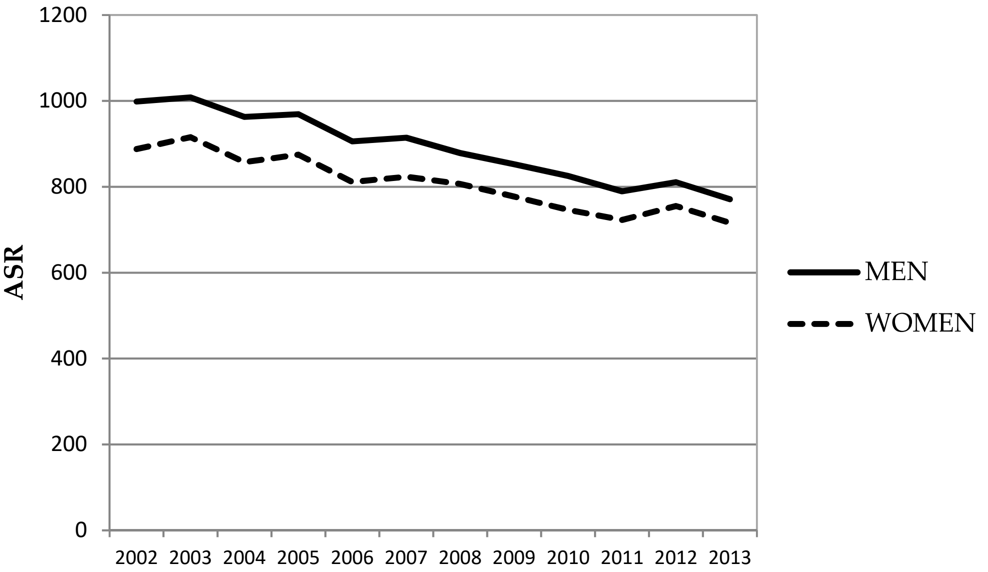

OVERALL MORTALITY
MENABLE MORTALITY

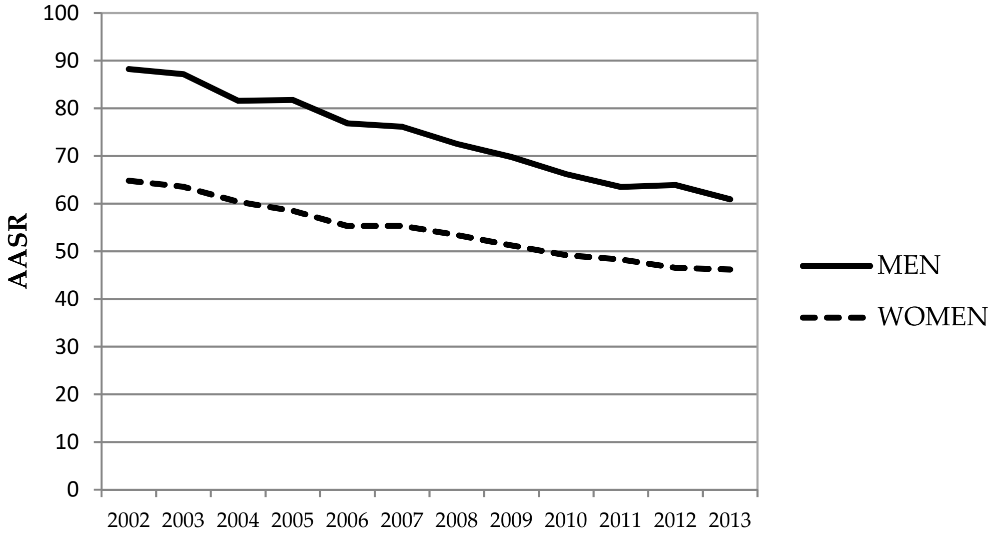

Supp1ementary Figure S1. Overall and amenable mortality annual standardised rates for the study period in Spain (2002-2013).

Supplementary Table S3. Standardised mortality rates, relative decreases, and 95% confidence intervals, by province. Periods 2002-07 and 2008-13. Men.

PROVINCE	ITV	ASR[$] 2002-07	ASR[$] 2008-13	Δ ASR[#]	95%CI (Δ)	AASR[$] 2002-07	AASR[$] 2008-13	Δ AASR[#]	95%CI (Δ)
Alava	-0.60	891.75	771.36	13.50	10.69 - 16.22	67.63	54.84	18.91	9.17 - 27.61
Albacete	0.47	871.90	775.75	11.03	8.56 - 13.43	68.50	55.89	18.41	9.44 - 26.49
Alicante	0.80	902.47	731.40	18.96	17.86 - 20.04	83.58	64.51	22.82	19.46 - 26.04
Almeria	0.87	1,063.27	890.98	16.20	14.23 - 18.13	93.33	73.75	20.98	14.81 - 26.70
Asturias	-0.33	1,034.68	905.34	12.50	11.24 - 13.74	92.54	77.33	16.44	12.08 - 20.57

Table S3. *Cont.*

Avila	0.53	860.39	750.40	12.78	9.58 - 15.87	77.17	61.80	19.92	7.76 - 30.48
Badajoz	-1.07	1,045.99	918.05	12.23	10.50 - 13.93	94.43	79.11	16.22	10.48 - 21.60
Balears (Illes)	0.47	943.39	819.30	13.15	11.55 - 14.73	72.88	58.80	19.32	13.94 - 24.36
Barcelona	0.73	961.70	811.98	15.57	14.92 - 16.21	74.38	61.22	17.69	15.43 - 19.89
Burgos	0.47	861.61	768.08	10.86	8.50 - 13.15	71.20	61.84	13.15	4.21 - 21.25
Caceres	-0.53	926.46	823.76	11.09	8.89 - 13.22	84.80	69.43	18.12	10.65 - 24.98
Cadiz	0.53	1,140.38	997.28	12.55	11.06 - 14.02	105.76	84.82	19.80	15.66 - 23.74
Cantabria	0.13	971.09	840.52	13.45	11.57 - 15.28	78.00	67.39	13.60	6.63 - 20.05
Castellón	1.27	981.74	831.90	15.26	13.34 - 17.15	81.98	70.27	14.28	7.33 - 20.72
Ceuta	-0.40	1,091.81	993.77	8.98	2.18 - 15.31	111.93	95.15	14.99	-3.45 - 30.15
Ciudad Real	0.47	956.64	822.71	14.00	12.02 - 15.94	79.35	63.54	19.92	12.76 - 26.50
Cordoba	0.00	1,022.38	883.74	13.56	11.92 - 15.17	89.07	72.88	18.18	12.70 - 23.31
Coruña (A)	-0.47	972.05	848.34	12.73	11.42 - 14.02	82.49	68.62	16.81	12.35 - 21.05
Cuenca	-0.07	812.60	744.70	8.36	5.20 - 11.41	67.31	64.68	3.91	-9.64 - 15.78
Girona	0.60	905.12	784.59	13.32	11.48 - 15.11	69.73	58.53	16.06	9.35 - 22.27
Granada	0.20	1,025.66	888.55	13.37	11.78 - 14.93	93.43	74.62	20.13	15.13 - 24.84
Guadalajara	0.47	770.14	681.12	11.56	8.11 - 14.87	64.40	52.23	18.90	5.95 - 30.06
Guipuzcoa	-1.20	965.96	820.57	15.05	13.32 - 16.75	78.99	60.62	23.26	17.68 - 28.45
Huelva	-0.27	1,113.95	973.86	12.58	10.43 - 14.67	101.57	86.39	14.95	8.11 - 21.27
Huesca	-0.13	868.90	751.05	13.56	10.72 - 16.31	71.11	62.97	11.45	-0.41 - 21.91
Jaen	-0.07	1006.68	900.00	10.60	8.79 - 12.37	89.84	75.32	16.16	10.09 - 21.82
Leon	0.27	881.89	771.17	12.55	10.66 - 14.41	74.38	62.37	16.15	8.93 - 22.79
Lleida	0.67	930.38	799.91	14.02	11.87 - 16.13	71.81	57.87	19.41	11.07 - 26.97
Lugo	-1.13	913.88	825.68	9.65	7.49 - 11.77	87.34	73.46	15.89	8.15 - 22.98
Madrid	0.00	894.88	736.63	17.68	17.03 - 18.34	70.35	53.60	23.81	21.66 - 25.90
Malaga	0.73	1,022.83	843.22	17.56	16.33 - 18.77	96.14	75.55	21.42	17.75 - 24.92
Melilla	-0.67	1,085.16	967.21	10.87	3.62 - 17.57	94.62	90.44	4.42	-20.35 - 24.09
Murcia	1.07	1,003.61	852.97	15.01	13.65 - 16.35	86.37	69.52	19.51	15.19 - 23.61
Navarra	-0.47	882.78	747.57	15.32	13.42 - 17.17	68.97	53.75	22.07	15.33 - 28.27
Ourense	-1.07	895.06	797.38	10.91	8.69 - 13.08	75.97	70.86	6.73	-2.49 - 15.12
Palencia	-0.93	946.55	817.15	13.67	10.51 - 16.72	83.55	71.05	14.96	2.96 - 25.48
Palmas (Las)	0.07	1,031.04	860.43	16.55	14.88 - 18.19	95.47	70.46	26.20	21.67 - 30.46
Pontevedra	-0.27	963.76	839.02	12.94	11.41- 14.45	76.36	61.43	19.55	14.38 - 24.41
Rioja (la)	0.53	894.70	778.99	12.93	10.30 - 15.49	75.14	62.63	16.65	7.10 - 25.22
Salamanca	-0.40	804.13	701.44	12.77	10.41 - 15.07	68.89	56.83	17.51	8.56 - 25.58
S.C.de Tenerife	0.07	938.12	795.81	15.17	13.52 - 16.79	88.96	66.34	25.43	20.75 - 29.83
Segovia	0.40	820.33	706.77	13.84	10.31 - 17.24	61.83	52.96	14.35	-0.86 - 27.26
Sevilla	-0.33	1,122.33	973.43	13.27	12.11 - 14.41	112.20	87.97	21.60	18.43 - 24.64
Soria	-0.53	776.43	691.51	10.94	6.38 - 15.27	63.65	51.97	18.35	-0.36 - 33.58
Tarragona	0.73	945.99	811.66	14.20	12.49 - 15.88	76.18	63.59	16.53	10.40 - 22.23
Teruel	-0.20	824.12	746.43	9.43	5.76 - 12.95	75.70	57.79	23.66	10.76 - 34.69
Toledo	0.87	880.92	749.32	14.94	13.08 - 16.75	67.19	52.89	21.28	14.35 - 27.66
Valencia	1.07	1,043.29	888.95	14.79	13.86 - 15.72	91.12	73.95	18.84	15.86 - 21.72
Valladolid	0.00	901.99	771.30	14.49	12.46 - 16.47	75.32	62.36	17.21	10.08 - 23.77
Vizcaya	-0.87	994.25	845.57	14.95	13.64 - 16.25	83.95	67.57	19.51	15.18 - 23.62
Zamora	-0.93	806.58	734.57	8.93	5.87 - 11.88	72.80	64.89	10.87	-1.18 - 21.48
Zaragoza	0.53	955.61	821.40	14.04	12.60 - 15.46	81.49	65.64	19.45	14.45 - 24.16

$ ASR = Overall mortality age standardised rate. AASR = Amenable mortality age standardised rate. Standard population: Population: The sum of the populations during the period 2002-13. # Δ ASR = Percentage decrease in ASR between 2002-07 and 2008-13. Δ AASR = Percentage decrease in AASR between 2002-07 and 2008-13. IV = Total vulnerability index by province.

Supplementary Table S4. Standardised mortality rates, relative decreases, and 95% confidence intervals, by province. Periods 2002-07 and 2008-13. Women.

PROVINCE	ITV	ASR[$] 2002-07	ASR[$] 2008-13	Δ ASR[#]	95%CI (Δ)	AASR[$] 2002-07	AASR[$] 2008-13	Δ AASR[#]	95%CI (Δ)
Alava	-0.60	772.06	671.39	13.04	10.02 - 15.95	42.07	41.22	2.02	-12.57 - 14.72
Albacete	0.47	867.63	769.07	11.36	8.81 - 13.84	53.14	43.94	17.31	6.94 - 26.53
Alicante	0.80	881.71	735.51	16.58	15.37 - 17.78	60.82	48.89	19.62	15.48 - 23.55
Almeria	0.87	989.45	866.70	12.41	10.18 - 14.58	74.66	55.12	26.17	19.56 - 32.24
Asturias	-0.33	863.26	767.89	11.05	9.75 - 12.33	59.80	51.02	14.68	9.27 - 19.77
Avila	0.53	771.76	702.36	8.99	5.52 - 12.34	65.26	46.01	29.50	16.77 - 40.28
Badajoz	-1.07	947.77	838.61	11.52	9.70 - 13.30	70.32	57.32	18.49	11.96 - 24.53
Balears (Illes)	0.47	871.61	790.24	9.34	7.59 - 11.05	58.24	48.58	16.59	10.38 - 22.36
Barcelona	0.73	839.75	731.42	12.90	12.22 - 13.57	53.94	45.60	15.46	12.82 - 18.02
Burgos	0.47	722.82	655.70	9.29	6.73 - 11.77	50.87	43.00	15.47	4.64 - 25.07
Caceres	-0.53	829.97	732.21	11.78	9.49 - 14.01	59.58	51.33	13.85	4.38 - 22.37
Cadiz	0.53	1,049.42	932.44	11.15	9.55 - 12.72	80.07	65.92	17.67	12.87 - 22.21
Cantabria	0.13	795.46	712.20	10.47	8.46 - 12.43	52.42	45.65	12.91	4.43 - 20.64
Castellón	1.27	927.00	792.59	14.50	12.46 - 16.49	62.76	47.96	23.58	16.17 - 30.34
Ceuta	-0.40	1,099.18	1,024.91	6.76	-0.54 - 13.53	94.68	79.99	15.52	-4.93 - 31.98
Ciudad Real	0.47	937.65	809.34	13.68	11.68 - 15.64	62.95	53.09	15.66	7.44 - 23.15
Cordoba	0,00	932.29	798.24	14.38	12.70 - 16.02	64.27	53.54	16.70	10.28 - 22.65
Coruña (A)	-0.47	841.50	761.23	9.54	8.17 - 10.89	57.87	49.64	14.22	8.87 - 19.26
Cuenca	-0.07	791.54	721.20	8.89	5.68 - 11.99	49.54	43.52	12.15	-2.93 - 25.02
Girona	0.60	841.96	732.08	13.05	11.12 - 14.94	50.96	43.03	15.56	7.49 - 22.92
Granada	0.20	977.46	853.88	12.64	10.96 - 14.29	70.50	57.48	18.47	12.68 - 23.87
Guadalajara	0.47	756.17	655.23	13.35	9.82 - 16.74	50.32	39.03	22.44	7.68 - 34.83
Guipuzcoa	-1.20	793.05	693.94	12.50	10.65 - 14.31	47.79	39.98	16.34	8.69 - 23.35
Huelva	-0.27	1,014.53	924.69	8.86	6.55 - 11.11	81.70	67.18	17.77	10.37 - 24.57
Huesca	-0.13	799.25	720.10	9.90	6.82 - 12.88	49.99	45.16	9.66	-5.38 - 22.55
Jaen	-0.07	967.69	840.94	13.10	11.24 - 14.91	64.97	53.35	17.89	10.96 - 24.27
Leon	0.27	757.52	676.48	10.70	8.70 - 12.65	51.62	46.80	9.34	0.13 - 17.69
Lleida	0.67	876.47	759.61	13.33	11.07 - 15.54	57.09	47.58	16.66	6.96 - 25.35
Lugo	-1.13	800.68	710.56	11.26	9.10 - 13.36	58.75	44.83	23.69	14.72 - 31.73
Madrid	0,00	777.30	666.78	14.22	13.53 - 14.90	52.28	41.49	20.64	18.19 - 23.01
Malaga	0.73	969.57	825.51	14.86	13.51 - 16.19	73.17	55.64	23.96	19.86 - 27.84
Melilla	-0.67	1,104.90	917.48	16.96	10.14 - 23.27	84.72	73.15	13.66	-10.16 - 32.33
Murcia	1.07	973.62	837.52	13.98	12.53 - 15.40	65.62	53.72	18.13	13.13 - 22.85
Navarra	-0.47	757.61	676.14	10.75	8.69 - 12.77	44.22	36.53	17.39	8.45 - 25.46
Ourense	-1.07	774.90	712.58	8.04	5.77 - 10.26	52.89	47.80	9.62	-1.24 - 19.32
Palencia	-0.93	765.93	702.26	8.31	4.83 - 11.67	58.48	51.81	11.41	-3.87 - 24.43
Palmas (Las)	0.07	940.26	814.89	13.33	11.40 - 15.23	72.60	54.65	24.72	19.38 - 29.71
Pontevedra	-0.27	820.15	737.59	10.07	8.48 - 11.62	55.43	47.40	14.49	8.31 - 20.25
Rioja (La)	0.53	780.04	697.16	10.63	7.77 - 13.39	48.58	46.01	5.29	-8.13 - 17.05
Salamanca	-0.40	737.80	649.20	12.01	9.61 - 14.34	55.86	44.04	21.16	11.47 - 29.79
S.C.de Tenerife	0.07	867.15	774.40	10.70	8.82 - 12.54	64.08	54.42	15.07	9.02 - 20.73
Segovia	0.40	731.18	683.80	6.48	2.56 - 10.24	54.36	50.28	7.51	-10.33 - 22.45
Sevilla	-0.33	1,018.39	890.23	12.58	11.39 - 13.76	77.78	62.93	19.09	15.30 - 22.71
Soria	-0.53	719.62	632.16	12.15	7.58 - 16.50	43.06	43.71	-1.51	-20.78 - 30.07
Tarragona	0.73	897.78	769.05	14.34	12.53 - 16.11	58.24	48.31	17.05	9.93 - 23.61
Teruel	-0.20	779.71	699.31	10.31	6.54 - 13.93	54.84	46.70	14.84	-2.60 - 29.32
Toledo	0.87	870.74	726.10	16.61	14.73 - 18.45	56.44	44.44	21.26	13.58 - 28.26
Valencia	1.07	945.80	821.80	13.11	12.13 - 14.08	65.44	53.76	17.85	14.36 - 21.20
Valladolid	0,00	797.20	703.32	11.78	9.61 - 13.89	56.85	46.26	18.63	10.50 - 26.02
Vizcaya	-0.87	826.36	712.69	13.76	12.37 - 15.12	55.16	46.07	16.48	11.05 - 21.57
Zamora	-0.93	751.12	673.59	10.32	7.30 - 13.25	54.69	44.88	17.94	4.30 - 29.63
Zaragoza	0.53	860.98	745.18	13.45	11.96 - 14.92	59.76	47.70	20.18	14.41 - 25.56

$ ASR = Overall mortality age standardised rate. AASR = Amenable mortality age standardised rate. Standard population: Population: The sum of the populations during the period 2002-13. # Δ ASR = Percentage decrease in ASR between 2002-07 and 2008-13. Δ AASR = Percentage decrease in AASR between 2002-07 and 2008-13. IV = Total vulnerability index by province.
